# Effect of Goal-Directed Crystalloid versus Colloid Administration on Perioperative Hemostasis in Partial Hepatectomy: A Randomized, Controlled Trial

**DOI:** 10.3390/jcm10081651

**Published:** 2021-04-13

**Authors:** Johannes Gratz, Oliver Zotti, André Pausch, Marion Wiegele, Edith Fleischmann, Thomas Gruenberger, Claus G. Krenn, Barbara Kabon

**Affiliations:** 1 Department of Anaesthesia, Intensive Care Medicine and Pain Medicine, Medical University of Vienna, Spitalgasse 23, 1090 Vienna, Austria; johannes.gratz@meduniwien.ac.at (J.G.); oliver.zotti@meduniwien.ac.at (O.Z.); andre.pausch@meduniwien.ac.at (A.P.); marion.wiegele@meduniwien.ac.at (M.W.); claus.krenn@meduniwien.ac.at (C.G.K.); barbara.kabon@meduniwien.ac.at (B.K.); 2 Department of Surgery, HPB-Center, Kaiser Franz Josef Hospital Vienna, Kundratstrasse 3, 1100 Vienna, Austria; tgruenberger@icloud.com

**Keywords:** goal-directed fluid management, perioperative hemostasis, colloids, abdominal surgery, viscoelastic coagulation tests

## Abstract

The use of colloids may impair hemostatic capacity. However, it remains unclear whether this also holds true when colloids are administered in a goal-directed manner. The aim of the present study was to assess the effect of goal-directed fluid management with 6% hydroxyethyl starch 130/0.4 on hemostasis compared to lactated Ringer’s solution in patients undergoing partial hepatectomy. We included 50 patients in this prospective, randomized, controlled trial. According to randomization, patients received boluses of either hydroxyethyl starch or lactated Ringer’s solution within the scope of goal-directed fluid management. Minimum perioperative FIBTEM maximum clot firmness (MCF) served as the primary outcome parameter. Secondary outcome parameters included fibrinogen levels and estimated blood loss. In the hydroxyethyl starch (HES) group the minimum FIBTEM MCF value was significantly lower (effect size −6 mm, 95% CI −10 to −3, *p* < 0.001) in comparison to the lactated Ringer’s solution (RL) group. These results returned to normal within 24 h. We observed no difference in plasma fibrinogen levels (RL 3.08 ± 0.37 g L^−1^ vs HES 2.65 ± 0.64 g L^−1^, *p* = 0.18) or the amount of blood loss between the two groups (RL 470 ± 299 mL vs HES 604 ± 351 mL, *p* = 0.18). We showed that goal-directed use of HES impairs fibrin polymerization in a dose-dependent manner when compared with RL. Results returned to normal on the first postoperative day without administration of procoagulant drugs and no differences in blood loss were observed.

## 1. Introduction

The administration of intravenous fluid therapy is one of the most basic elements of perioperative care. Yet, it constitutes a key component of anesthesiological management that can directly affect patient outcomes. Colloids carry the theoretical advantage of remaining in the intravascular compartment longer than crystalloids, and it has thus been suggested that the administration of colloids might be useful for faster resuscitation in hypovolemic patients [1]. Furthermore, particularly in the context of goal-directed fluid therapy, a potentially beneficial volume-sparing effect of colloid administration has been proposed [2].

However, in spite of a lively scientific debate over the last two decades, the perioperative use of synthetic colloids remains a controversial issue [3,4,5,6,7]. Following two studies published in 2012 [8,9], the European Medicine Agency (EMA) and the US Food and Drug Administration (FDA) issued warnings that hydroxyethyl starch (HES) should no longer be used in critically ill patients due to a possible rise in kidney injury and mortality associated with the administration of HES [10,11]. However, these recommendations derived from studies performed in critically ill patients, and the EMA explicitly stated that HES could still be used perioperatively, e.g., in the case of acute hemorrhage. Three recently published large outcome trials showed no increase in a composite outcome of major postoperative complications with the perioperative use of HES in comparison with crystalloids [12,13,14].

Among a number of potential adverse effects, the administration of HES has been linked with an increased risk of acquired coagulopathy [15]. Impaired fibrin polymerization has been identified as the most prominent contributor to hemostatic derangement following the use of HES [16]. Among a number of other disadvantages when used in the perioperative setting, conventional coagulation tests, such as prothrombin time (PT), activated partial thromboplastin time (aPTT), and fibrinogen levels fail to sufficiently detect impaired fibrin polymerization [17]. In contrast, viscoelastic coagulation test methods, such as rotational thromboelastometry (ROTEM), allow the detection of impaired fibrin polymerization.

Of note, a large number of studies evaluating the influence of perioperatively administered HES on hemostasis were performed in cardiovascular surgery [3,15,18,19,20]. Due to the manifold influences on hemostasis and bleeding present in cardiovascular surgery, results from such studies cannot readily be transferred to other clinical settings. Furthermore, the influence of goal-directed administration of HES on hemostasis has not been sufficiently investigated thus far.

Therefore, this prospective, randomized, controlled trial was designed as a sub-study of a recently published large outcome trial to assess the effect of goal-directed administration of 6% HES 130/0.4 in comparison to lactated Ringer’s solution (RL) on hemostasis in major abdominal surgery [13]. Specifically, we tested the hypothesis that fibrin polymerization as measured by ROTEM might be reduced during goal-directed colloid administration compared to sole administration of crystalloids in patients undergoing open partial hepatectomy. Furthermore, we aimed to investigate the time course of this possible HES-associated impairment of fibrin polymerization. Secondary outcome parameters included results of conventional coagulation tests and estimated blood loss.

## 2. Materials and Methods

### 2.1. Study Design

The present investigator-initiated, prospective, randomized, controlled trial was performed at a single tertiary center (Medical University of Vienna, Vienna, Austria). The study was approved by the ethics committee of the Medical University of Vienna (EK 431/2005). It was conducted in accordance with the Declaration of Helsinki and Good Clinical Practice and registered at Clinicaltrials (NCT00517127, http://clinicaltrials.gov/ct2/show/NCT00517127 (accessed on 11 April 2021)) and EudraCT (2005-004602-86) before the inclusion of patients. Written informed consent was obtained from all patients before inclusion. The Consolidated Standards of Reporting Trials (CONSORT) guidelines were followed to report the results of the present study [21]. This study was conducted as an embedded sub-study of a large outcome trial evaluating the effects of goal-directed use of colloids and crystalloids on postoperative combined morbidity [13].

### 2.2. Study Participants

Fifty patients were assigned to one of two groups before induction of anesthesia; this was done using a reproducible set of computer-generated random codes, and patients were not informed of their group assignments:

- 25 patients in the **RL group** received repeated fluid boluses of 250 mL of lactated Ringer’s solution (Ringer-Lactat; Fresenius Kabi, Germany) when hypovolemia was detected.
- 25 patients in the **HES group** received repeated fluid boluses of 250 mL of 6% hydroxyethyl starch 130/0.4 (Voluven; Fresenius Kabi, Germany) in case of hypovolemia.

Fluid management was guided and hypovolemia detected by esophageal Doppler monitoring (Cardiac Q; Deltex Medical Group PLC, United Kingdom) according to a previously published algorithm [13,22].

Patients aged 18 to 80 years, with an American Society of Anesthesiologist (ASA) classification I-III, a body mass index (BMI) <35 kg m^−2^, and who were scheduled for elective open partial hepatectomy were eligible for inclusion in the study. Major resection was defined as resection of four or more liver segments, as previously described [23]. We excluded patients suffering from cardiac insufficiency (estimated cardiac ejection fraction less than 35%), renal insufficiency (estimated creatinine clearance less than 30 mL min^−1^), severe chronic obstructive pulmonary disease, coagulopathies, or known esophageal or aortic abnormalities.

### 2.3. Anesthetic Procedures

Before the induction of anesthesia, standard monitoring (ECG, noninvasive blood pressure, oxygen saturation) was established. Arterial and central venous lines were placed after induction of anesthesia with fentanyl (1–3 mcg kg^−1^), propofol (1–3 mg kg^−1^), and rocuronium (0.6 mg kg^−1^). Subsequently, anesthesia was maintained with sevoflurane and fentanyl. Patients were ventilated with tidal volumes between 8 and 10 mL kg^−1^ of ideal body weight, a peak inspiratory pressure below 30 mmHg, a positive end-expiratory pressure (PEEP) of 5 mmHg or higher, and a respiratory rate to maintain end-tidal carbon dioxide tension close to 35 mmHg. Ideal body weight was calculated according to the Robinson formula [24]. To maintain normothermia, patients were actively warmed with convective warming.

Patients were given 5–7 mL kg^−1^ of RL during anesthetic induction, followed by 3–5 mL kg^−1^ h^−1^ throughout the surgery. Patients in the HES group received boluses of 250 mL of 6% HES 130/0.4, whereas patients in the RL group received boluses of 250 mL of RL when hypovolemia was detected. For cases in which mean arterial pressure was <65 mmHg and there were no Doppler-based signs of hypovolemia, intravenous vasopressors were administered at the discretion of the attending anesthesiologist. During the postoperative study period, crystalloid was administered at 2 mL kg^−1^ h^−1^ with additional fluid boluses as deemed clinically necessary. Postoperative analgesia was provided by institutional routine.

### 2.4. Measurements and Outcome Parameters

ROTEM is a viscoelastic method that allows real-time assessment of clot formation and dissolution in whole blood [25]. EXTEM, INTEM, and FIBTEM are commercially available ROTEM tests that refer to abnormalities in the extrinsic and intrinsic system, as well as fibrinogen polymerization and fibrinolysis. These tests, as well as PT, aPTT, plasma fibrinogen levels, and blood count, were measured preoperatively, after every 500 mL of fluid bolus (i.e., every second bolus of RL or HES), at the end of the surgery, and 24 h postoperatively. Blood samples were drawn from the arterial lines into tubes containing trisodium citrate 3.8% for ROTEM and conventional coagulation tests and containing potassium EDTA for blood cell counts (both Vacuette, Greiner, Kremsmuenster, Austria). ROTEM assays (Tem GmbH, Munich, Germany) were performed according to the manufacturer’s recommendations, and clotting time (CT) and maximum clot firmness (MCF) were analyzed.

Conventional coagulation tests were performed at the hospital’s central laboratory. PT, aPTT, and plasma fibrinogen levels were measured using an automated coagulation analyzer (STA-R, Evolution coagulometer, Diagnostica Stago S.A.S., Asnières sur Seine, France). Complete blood cell counts were measured with a Sysmex XE-2100 cell counter (Sysmex, Kobe, Japan).

The primary outcome parameter for this study was the perioperative minimum value of FIBTEM MCF (i.e., the minimum value of FIBTEM MCF during the study period). Secondary outcome parameters included summary measures of median FIBTEM MCF values and fibrinogen concentrations over the entire study period as well as over the intraoperative period. Furthermore, we evaluated estimated blood loss by direct measurement of blood in the suction unit, conventional coagulation tests, and ROTEM results other than the above-mentioned, and the administration of procoagulant drugs. Demographic and morphometric data, ASA physical status, as well as type and duration of surgery were recorded. We measured core temperature at the distal esophagus throughout surgery. Blood gas analyses were performed hourly.

### 2.5. Statistical Analysis

Sample size calculation was based on the expected difference in the primary outcome parameter of minimum perioperative FIBTEM MCF. Under the assumption of a difference of 25% between the groups and a standard deviation of 25% a sample size of 21 patients in each group was calculated, allowing a type I error of 5% and a type II error of 20%. To compensate for potential drop-outs, we decided to include 25 patients per group. Normality of the data distribution was tested using the Kolmogorov-Smirnov test. Normally distributed data are presented as mean ± SD. Those distributed otherwise are given as median (IQR). For continuous variables, the unpaired t-test or Mann-Whitney test were used as appropriate according to data distribution. The chi-square test was used for comparing categorical variables. We compared minimum values throughout the study period and summary measures of the intraoperative period as well as the entire study period (i.e., including measurements on the first postoperative day) and measurements on the first postoperative day. Two-sided *p*-values of <0.05 were accepted as statistically significant. GraphPad Prism 8.2 (GraphPad Software; San Diego, CA, USA) was used for statistical analyses and the production of graphs.

## 3. Results

### 3.1. Baseline Characteristics

**Figure 1:**
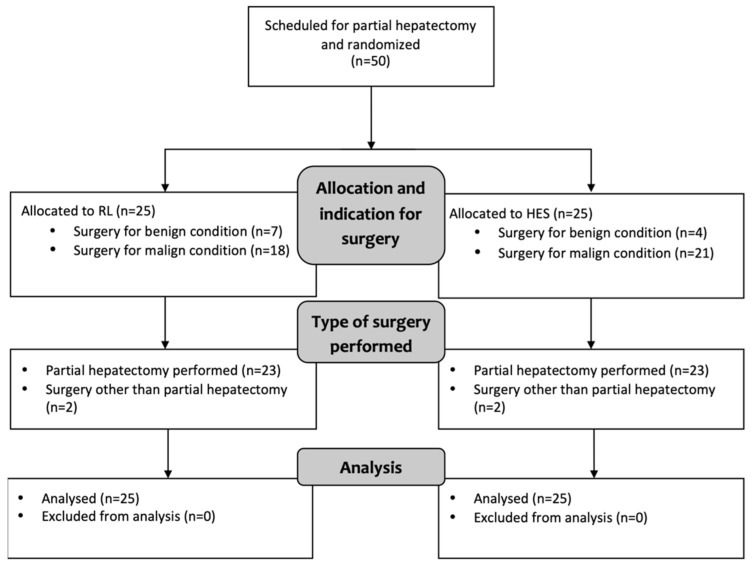
CONSORT flowchart depicting inclusion, randomization, and type of surgery (HES, hydroxyethyl starch; RL, Ringer’s lactate).

A total of 50 patients were randomly assigned to one of the two study groups. Twenty-five patients received HES, whereas RL was used in the control group of 25 patients. Figure 1 shows a flowchart depicting the inclusion and randomization of patients, as well as the indication for and type of surgery. Two patients in each study group did not undergo partial hepatectomy: one patient in each group underwent extended cholecystectomy, whereas another patient in each group underwent exploratory laparotomy with multiple open liver biopsies. None of the patients in either study group underwent vascular resection and reconstruction.

Demographic data, duration of surgery, and preoperative baseline laboratory values were comparable between the groups (Table 1). Intraoperative characteristics, including overall fluid balance, and confounding factors with regard to hemostasis, such as temperature, pH, and calcium levels, did not differ between groups (Table 2). In order to exclude differences in the preoperative degree of hepatic dysfunction we calculated model of endstage liver disease (MELD) scores that were comparably low in both groups (RL 7 ± 2 vs. HES 7 ± 2 mm, *p* = 0.79).

### 3.2. ROTEM Measurements and Plasma Fibrinogen Levels

**Figure 2:**
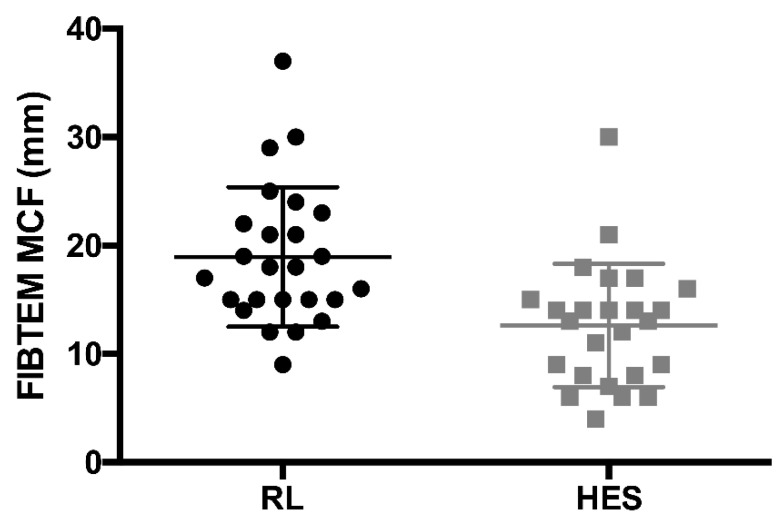
Difference in perioperative minimum of FIBTEM MCF: RL 19 ± 6 mm vs. HES 13 ± 6 mm; *p* < 0.001 (HES, hydroxyethyl starch; MCF, maximum clot firmness; RL, Ringer’s lactate).

**Figure 3:**
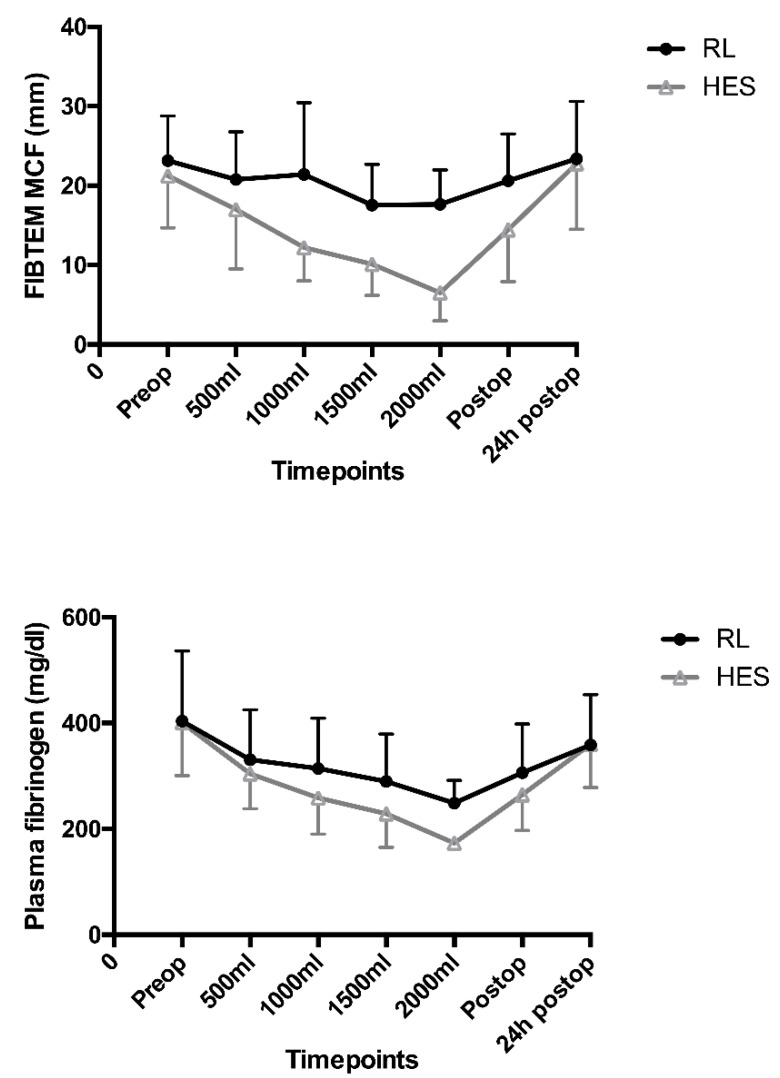
Time course of FIBTEM MCF results and plasma fibrinogen levels preoperatively (RL/HES: *n* = 25) and after 500 mL (RL: *n* = 23, HES: *n* = 24), 1000 mL (RL: *n* = 20, HES: *n* = 18), 1500 mL (RL: *n* = 11, HES: *n* = 8), and 2000 mL (RL: *n* = 6, HES: *n* = 2) of study fluid, respectively, as well as immediately postoperatively and 24 h postoperatively (RL/HES: *n* = 25). (HES, hydroxyethyl starch; MCF, maximum clot firmness; RL, Ringer’s lactate).

The primary study endpoint of minimum perioperative FIBTEM MCF was significantly reduced in patients receiving HES, with an effect size of −6 mm (95% confidence interval (CI) −10 to −3, *p* < 0.001) (Figure 2) in comparison with the RL group. Figure 3 shows the progressive, dose-dependent decline of FIBTEM MCF over time, which was much more pronounced in the HES group in comparison with the RL group, depicting measurements after every second bolus (i.e., 500 mL) of the study fluid. Summary measures of FIBTEM MCF results over time were different when comparing the two groups throughout the intraoperative period (RL 20 ± 2 mm vs. HES 12 ±4 mm, *p* = 0.005) as well as throughout the entire study period (Table 3). However, FIBTEM MCF results in the HES group returned to normal 24 h after surgery without substitution of fibrinogen in any patient, and there was no significant difference in FIBTEM MCF after 24 h between the two groups (Table 3).

In contrast to the FIBTEM MCF results, summary measures of EXTEM MCF showed no significant difference throughout the intraoperative period (RL 64 ± 1 mm vs. HES 61 ± 5 mm, *p* = 0.13) or throughout the entire study period (Table 3). Summary measures of EXTEM CT values were different between the two groups throughout the intraoperative period (RL 56 ± 2 s vs. HES 70 ± 8 s, *p* = 0.004), as well as throughout the entire study period (Table 3). Other ROTEM results, including INTEM tests, remained within the normal range throughout the study period, without significant differences between the two groups (Table 3). Plasma fibrinogen levels showed similar changes over time as FIBTEM MCF results (Figure 3). However, these changes were much less pronounced, and when comparing summary measures throughout the intraoperative period (RL 2.98 ± 0.31 g L^−1^ vs. HES 2.46 ± 0.49 g L^−1^; *p* = 0.08) as well as throughout the entire study period, no significant differences were observed (Table 3). A comparison of the minimum plasma fibrinogen levels throughout the study period did not show a difference either (RL 2.92 ± 0.92 g dL^−1^ vs. HES 2.49 ± 0.67 g L^−1^ *p* = 0.07).

### 3.3. Conventional Coagulation Tests

aPTT values remained within the reference range throughout the study period. However, there was a statistically significant difference between the two groups when comparing summary measures with slightly increased aPTT values in the HES group (Table 3). PT decreased marginally below the reference range 24 h postoperatively in the RL as well as the HES group without a significant difference between the two groups, whereas summary measures remained within the normal range (Table 3).

### 3.4. Estimated Blood Loss, Cell Counts, and Procoagulant Medication

There was no statistically significant difference in blood loss between the two groups (Table 2). Hemoglobin levels slightly decreased intraoperatively, with their lowest values at 109 ± 15 g L^−1^ in the RL group and 94 ± 7 g L^−1^ in the HES group (*p* = 0.18). However, a comparison of summary measures between the two groups showed no statistically significant difference (Table 3). Platelet counts also decreased over time, with a significant difference between the two groups when comparing the summary measures throughout the intraoperative period (RL 202 ± 20 G L^−1^ vs. HES 149 ± 26 G L^−1^; *p* = 0.007) as well as throughout the study period (Table 3). In the RL group, no allogenic blood products were transfused, whereas two patients in the HES group received one unit of packed red blood cells each. One patient in the RL group received 500 IU prothrombin complex concentrate intraoperatively at the discretion of the attending anesthesiologist. None of the other patients in either group received any hemostatic therapy.

## 4. Discussion

In the present study, we demonstrated that the goal-directed use of 6% HES 130/0.4 impaired viscoelastic whole-blood coagulation measurements in adults undergoing partial hepatectomy in a dose-dependent manner. In comparison with RL, the administration of HES led to significantly lower FIBTEM MCF values and prolonged EXTEM CT values. Of note, this dose-dependent coagulation impairment was of a self-limiting nature, and ROTEM results spontaneously returned to values within the normal range on the first postoperative day without the administration of procoagulant drugs.

In reference to the perioperative use of colloid solutions, a number of mechanisms leading to disturbances in coagulation have been proposed, including a decrease in factor VIII, antiplatelet effects, and a profibrinolytic effect [16,26]. Some of these mechanisms were found to be much more pronounced in the older high- and medium-molecular-weight HES solutions [27,28]. In contrast, impaired fibrin polymerization, being the most important contributor to colloid-induced coagulopathy, holds true for all HES solutions; thus also including the examined low-molecular-weight 6% HES 130/0.4 [16]. In line with this, we observed a significant decrease of FIBTEM MCF values in the HES group, whereas there was no difference in EXTEM MCF values between the two groups in spite of a reduced platelet count in the HES group. With regard to the difference in FIBTEM MCF between the two study groups, it is noteworthy that we observed no difference in plasma fibrinogen levels between the groups. The observation of a more pronounced decrease in FIBTEM MCF when compared with plasma fibrinogen levels after administration of HES has been described in vitro before [29]. We could reproduce this in a clinical setting, reflecting the functional nature of viscoelastic whole-blood assays in comparison with conventional coagulation tests.

Our study revealed prolonged EXTEM CT values in the HES group despite of no differences in PT between the groups. Although EXTEM CT values primarily represent clot initiation dynamics, a relevant influence of the substrate fibrinogen on EXTEM CT has been described before [30,31]. Consequently, current guidelines for the management of major bleeding recommend further procoagulant medication only once fibrinogen levels, as represented by FIBTEM MCF, have been corrected [32]. In light of this, prolonged EXTEM CT values in the present study can be interpreted as reflecting the functional fibrinogen deficiency caused by impaired fibrin polymerization. An earlier study that evaluated the influence of goal-directed administration of HES 130/0.4 on hemostasis in abdominal surgery employed thrombelastography to characterize hemostatic changes [33]. In contrast to our study, the authors did not report prolonged clotting times, whereas they also reported reduced clot strength. Although thromboelastometry and thrombelastrography are similar viscoelastic methods, the obtained values (e.g., CT for thromboelastometry and r time for thrombelastography) are not readily comparable, which might explain the different findings.

Our main finding of impaired clot strength corroborates similar results in a number of studies and systematic reviews that have previously been published [19,20,26,28,34,35,36,37]. Randomized, controlled trials have shown impaired coagulation competence with the use of HES 130/0.4 in cardiovascular surgery as assessed by ROTEM [19,20]. Yet, some studies in the cardiovascular setting have shown contradicting results, with the use of HES leading to no relevant impairment of coagulation competence [18,38]. It seems noteworthy that cardiovascular surgery is a field with a particularly high number of diverse impacts on perioperative hemostasis and bleeding, such as the intraoperative use of extracorporeal circulation and the resulting need for anticoagulation. Results from studies carried out in this setting might thus not readily be transferrable to patients undergoing major abdominal surgery. Consequently, the present study was performed in patients undergoing open partial hepatectomy. Additionally to representing major abdominal surgery, the avoidance of transfusion of blood products is of particular interest in hepato-pancreato-biliary surgery, including liver resection, not least in terms of cancer recurrence [39].

When weighing our study against other randomized, controlled trials comparing the influence on hemostasis between 6% HES 130/0.4 and crystalloids in patients undergoing non-cardiac surgery, it needs to be recognized that the administration of HES was not goal-directed in most of the previously published studies [36,37,40,41,42]. Consequently, dilutional effects of fluid administration could have contributed to the previously reported results. In our study, the only slight reduction of plasma fibrinogen levels together with relatively stable blood hemoglobin concentrations throughout the study period showed that the impact of HES on hemostasis could not solely be explained by dilutional effects. Dilutional effects might also play a relevant role when examining blood loss as an outcome parameter. Increased blood loss has been described with the use of HES in a systematic review and meta-analysis [15]. However, none of the studies included in this meta-analysis evaluated the use of HES within a goal-directed regimen, thus avoiding unnecessary iatrogenic hyperhydration and unphysiological hemodilution. It needs to be stressed that the present work was not intended to detect a difference in blood loss. However, we investigated estimated blood loss as a secondary outcome parameter and did not observe a difference between the two study groups. Furthermore, the present work was performed as an embedded sub-study within a large clinical outcome trial that showed no increase in blood loss or transfusion requirements with the use of HES [13]. This is in line with another recently published outcome trial that compared the use of HES and crystalloids in 775 patients undergoing major abdominal surgery and found no difference in blood loss between the groups [12].

Relevant limitations of our study need to be recognized. Importantly, although we observed statistically significant differences in viscoelastic whole-blood coagulation assays, the clinical relevance of our findings remains to be determined. In the present work, neither FIBTEM MCF nor EXTEM CT values reached critical thresholds that would have led to substitution of factor concentrates in a bleeding patient according to current guidelines [32,43]. Secondly, our study was not designed and powered to detect a difference in clinically relevant endpoints, such as blood loss or the amount of blood products used. A third limitation of our study is that we did not investigate colloids other than HES. Data on the influence of human albumin and gelatin on hemostasis exist and have suggested a similar or lower risk of impaired coagulation competence in comparison with HES solutions [3,19,20,26,35]. However, it remains debatable whether other colloidal substances might have less influence on hemostasis than HES.

## 5. Conclusions

The present study demonstrated that goal-directed fluid management with 6% HES 130/0.4 impaired viscoelastically determined whole-blood coagulation parameters in adult patients undergoing open partial hepatectomy in a dose-dependent manner. FIBTEM MCF and EXTEM CT values showed changes compatible with impaired fibrin polymerization. However, values returned to normal within 24 h without the administration of procoagulant drugs, and the observed changes did not translate into a significant difference in the clinically relevant outcome parameter of blood loss.

## Figures and Tables

**Table 1:** Patient characteristics, operative data, and baseline laboratory values. Values are number of patients or mean ± SD.

	RL (*n* = 25)	HES (*n* = 25)
Gender (male/female)	13/12	15/10
Age (years)	56 ± 16	58 ± 12
BMI (kg m^−2^)	25 ± 4	26 ± 6
ASA status (I/II/III)	5/14/6	6/8/11
Liver resection (minor/major)	14/9	19/4
Duration of surgery (min)	212 ± 79	228 ± 86
Preoperative chemotherapy	10	12
FIBTEM MCF (mm)	23 ± 6	21 ± 7
EXTEM CT (s)	57 ± 11	60 ± 10
EXTEM MCF (mm)	66 ± 7	66 ± 6
INTEM CT (s)	156 ± 53	152 ± 35
INTEM MCF (mm)	67 ± 6	68 ± 4
PT (%)	103 ± 27	104 ± 21
aPTT (s)	34.5 ± 4.9	36.4 ± 4.3
Plasma fibrinogen (g L^−1^)	4.04 ± 1.32	4.01 ± 1
Hemoglobin (g L^−1^)	127 ± 13	128 ± 20
Platelet count (G L^−1^)	247 ± 91	231 ± 98

ASA status, American Society of Anesthesiologists physical status classification; aPTT, activated partial thromboplastin time; BMI, body mass index; HES, hydroxyethyl starch; PT, prothrombin time; RL, Ringer’s lactate.

**Table 2:** Intraoperative variables: fluid volumes, possible confounding factors to hemostasis, and estimated blood loss. Values are median (IQR) or mean ± SD as appropriate.

	RL (*n* = 25)	HES (*n* = 25)	*p*
Total fluid volume (L)	2.95 (1.55)	2.9 (1.57)	0.47
Crystalloid volume (L)	2.95 (1.55)	1.66 (0.87)	**<0.001**
Colloid volume (L)	-	1.12 ± 0.49	-
Lowest temperature (°C)	35.9 ± 0.5	35.9 ± 0.5	0.82
Lowest pH	7.31 ± 0.03	7.32 ± 0.04	0.42
Lowest Ca^2+^ (mmol L^−1^)	1.16 ± 0.05	1.16 ± 0.05	0.53
Estimated blood loss (mL)	470 ± 299	604 ± 351	0.16

HES, hydroxyethyl starch; RL, Ringer’s lactate.

**Table 3:** Summary measures throughout the entire study period and 24 h postoperative results of ROTEM, conventional coagulation tests, plasma fibrinogen levels, hemoglobin levels, and platelet counts. Values are mean ± SD.

	Summary Measures			24 h Postoperative		
	RL (*n* = 25)	HES (*n* = 25)	*p*	RL (*n* = 25)	HES (*n* = 25)	*p*
FIBTEM MCF (mm)	20 ± 2	14 ± 6	**0.03**	23 ± 7	23 ± 8	0.76
EXTEM CT (s)	57 ± 4	70 ± 7	**0.003**	60 ± 12	63 ± 7	0.15
EXTEM MCF (mm)	64 ± 1	60 ± 5	0.10	66 ± 6	64 ± 6	0.26
INTEM CT (s)	154 ± 10	155 ± 9	0.92	164 ± 48	152 ± 32	0.33
INTEM MCF (mm)	64 ± 2	62 ± 3	0.19	66 ± 5	67 ± 4	0.81
Plasma fibrinogen (g L^−1^)	3.08 ± 0.37	2.65 ± 0.64	0.18	3.59 ± 0.95	3.69 ± 0.82	0.95
PT (%)	78 ± 7	68 ± 13	0.09	63 ± 19	67 ± 17	0.35
aPTT (s)	34.8 ± 1.4	38.0 ± 2.9	**0.04**	37.2 ± 3.6	36.3 ± 3.8	0.43
Hemoglobin (g L^−1^)	111 ± 3	106 ± 12	0.34	107 ± 15	109 ± 14	0.53
Platelet count (G L^−1^)	202 ± 18	155 ± 28	**0.006**	201 ± 68	185 ± 53	0.38

aPTT, activated partial thromboplastin time; CT, clotting time; HES, hydroxyethyl starch; MCF, maximum clot firmness; PT, prothrombin time; RL, Ringer’s lactate; ROTEM, rotational thromboelastometry.

## Data Availability

The data presented in this study are available upon reasonable request from the corresponding author.
